# Generation and Characterization of Specific Antibodies to the Murine and Human Ectonucleotidase NTPDase8

**DOI:** 10.3389/fphar.2017.00115

**Published:** 2017-03-08

**Authors:** Julie Pelletier, Mabrouka Salem, Joanna Lecka, Michel Fausther, François Bigonnesse, Jean Sévigny

**Affiliations:** ^1^Centre de recherche du CHU de Québec – Université Laval, Québec CityQC, Canada; ^2^Département de Microbiologie-Infectiologie et d’Immunologie, Faculté de Médecine, Université Laval, Québec CityQC, Canada; ^3^Division of Gastroenterology and Hepatology, Department of Internal Medicine, University of Arkansas for Medical Sciences, Little RockAR, USA

**Keywords:** monoclonal antibodies, polyclonal antibodies, mouse NTPDase8, human NTPDase8, cDNA immunization

## Abstract

The ectonucleotidase nucleoside triphosphate diphosphohydrolase-8 (NTPDase8) is the last member of the Ecto-NTPDase family to be discovered and characterized. It is a transmembrane protein which regulates the concentration of the agonists of P1 and P2 receptors at the cell surface. The functions of the enzyme are still not known partly due to the lack of specific tools such as antibodies. In this work, guinea pig polyclonal antibodies against mouse NTPDase8 and mouse monoclonal antibodies against human NTPDase8 have been generated and characterized. For the production of antibodies against mouse NTPDase8 several techniques have been tried. Several peptide antigens in several hosts (rabbit, rat, hamster, and guinea pig) failed to give a positive reaction suggesting that NTPDase8 is poorly immunogenic. In this study, we describe the successful process that led to anti-mouse NTPDase8, namely the cDNA immunization technique. Monoclonal antibodies to human NTPDase8 were also obtained by cDNA immunization followed by a final injection with transfected human embryonic kidney (HEK 293T) cells expressing human NTPDase8. The specificity of these antibodies was evaluated by Western blot, immunocytochemistry, immunohistochemistry and flow cytometry. In contrast, all commercial antibodies to NTPDase8 peptides that we have tested failed to give a specific positive signal against the expressed NTPDase8 protein when used to probe Western blots. In addition, immunohistochemistry experiments confirmed the presence of NTPDase8 in mouse liver canaliculi. The tools generated in this work will help characterize NTPDase8 localization and function in future studies and its contribution to the modulation of P1 and P2 receptor activation.

## Introduction

The activation of nucleotide (P2) and adenosine (P1) receptors is regulated in part by enzymes that regulate the concentration of their agonists at the cell surface. The most important enzymes that dephosphorylate nucleotides in the extracellular environment in physiological conditions are members of the Ecto-nucleoside triphosphate diphosphohydrolase (E-NTPDase) family (Beaudoin et al., 1996; Robson et al., 2006; Zimmermann et al., 2012). This family of ectonucleotidases is composed of 8 members (NTPDase1 to -8; Robson et al., 2006; Zimmermann et al., 2012). NTPDase1, -2, -3, and -8 are expressed at the plasma membrane and they hydrolyse nucleotides at the cell surface with different abilities (Kukulski et al., 2005). These enzymes have been located in different systems and they have been reported to play distinct roles. To give a few examples, NTPDase1, which is expressed by several cell types which includes vascular endothelial cells and Tregs, has been shown to regulate vascular hemostasis and immune functions (Kukulski et al., 2011; Zimmermann et al., 2012; Yegutkin, 2014). NTPDase2, which is also expressed by several cell types, is found in type I cells of taste buds where it has been associated to taste functions (Bartel et al., 2006; Vandenbeuch et al., 2013). NTPDase3 has been detected in neurons in different organs. It was proposed that in the rat brain, NTPDase3 may modulate feeding and sleep–wake behavior (Belcher et al., 2005). In contrast to NTPDase1, -2, and -3, no function has yet been associated to NTPDase8. Finally, as NTPDase4, -5, -6, and -7 are mainly anchored to the membranes of intracellular organelles and as they hydrolyse nucleotides with lower affinities their functions are expected to differ from the one of the above plasma membrane bound NTPDases.

So far NTPDase8 was reported to be expressed only in a few tissues which includes rat (Fausther et al., 2007) and porcine (Sévigny et al., 2000) liver, and pig kidneys (Sévigny et al., 2000). The lack of tools such as antibodies limits the study of NTPDase8 structure and function. To date, some commercial antibodies against NTPDase8 are available, but their specificity has not been demonstrated. The goal of this work was to obtain specific antibodies against NTPDase8 and to demonstrate their specificity. As the commercial antibodies revealed to be unspecific, to achieve this goal we used several techniques of immunization. Following several unsuccessful attempts we finally ended with a convenient technique that we also describe here.

## Materials and Methods

### Materials

Aprotinin, phenylmethanesulfonyl fluoride, ethylenediamine tetraacetic acid, sodium citrate, paraformaldehyde (PFA), 3,3′-diaminobenzidine (DAB), and hydrogen peroxide (H_2_O_2_) were purchased from Sigma-Aldrich (Oakville, ON, Canada). Tris(hydroxymethyl)aminomethane (Tris) was from VWR International (Montreal, QC, Canada). Dulbecco’s modified Eagle’s medium and antibiotic–antimycotic solution, NuPAGE lithium dodecyl sulfate sample, NuPAGE 4–12% Bis-Tris gels were obtained from Life Technologies (Burlington, ON, Canada). Fetal bovine serum (FBS) and goat serum were from Wisent (St-Bruno, QC, Canada). For Western blot and/or immunohistochemistry experiments the secondary antibodies used were either conjugated to horseradish peroxidase (HRP), namely goat anti-guinea pig, donkey anti-goat (Santa Cruz Biotechnology, Dallas, TX, USA), goat anti-mouse (Jackson ImmunoResearch Laboratories Inc. West Grove, PA, USA), donkey anti-rabbit (GE Healthcare Life Sciences, Baie d’Urfe, QC, Canada), rabbit anti-rat (Thermo Fisher Scientific, Rockford, IL, USA), or to biotin, namely goat anti-guinea pig, goat anti-rabbit (Jackson ImmunoResearch Laboratories Inc. West Grove, PA, USA), goat anti-mouse and goat anti-rat (Vector Laboratories, Burlington, ON, Canada). For flow cytometry experiments Alexa Fluor 594-goat anti-guinea pig and Alexa Fluor 633-goat anti-mouse were obtained from Life Technologies (Burlington, ON, Canada).

### Animals and Plasmids

Female Sprague-Dawley rats, Hartley guinea pigs, LVG Golden Syrian hamsters, BALB/c mice, and New Zealand rabbits were obtained from Charles River Laboratories (Saint-Constant, QC, Canada). All procedures were approved by the Canadian Council on Animal Care and the Université Laval Animal Welfare Committee. The plasmids encoding mouse NTPDase1 (GenBank accession no. NM_009848; Enjyoji et al., 1999), mouse NTPDase2 (AY376711; Kukulski et al., 2005), mouse NTPDase3 (AY376710; Lavoie et al., 2004), mouse NTPDase8 (AY364442; Bigonnesse et al., 2004), human NTPDase1 (U87967; Kaczmarek et al., 1996), human NTPDase2 (NM_001246; Knowles and Chiang, 2003), a kind gift of Dr. A. F. Knowles (San Diego, CA, USA), human NTPDase3 (AF034840; Smith and Kirley, 1998), a kind gift of Dr. T. L. Kirley (Cincinnati, OH, USA), human NTPDase8 (AY430414; Fausther et al., 2007), or rat NTPDase8 (AY536920; Fausther et al., 2007) all in pcDNA 3.1 vector were used for antiserum generation and for/or cell transfection, as described below.

### Polyclonal Antibody Production to Mouse NTPDase8 with Peptides and Recombinant Proteins Produced in Bacteria

High density multiple antigen peptides (MAPs) system generated using four lysine residues bearing four branching peptides were synthesized separately with two polypeptides: peptide 829 (MGLSWKERVFMALL) and peptide 830 (QWPANKEKDTGVVSQ) that correspond to amino acid 1–14 and 60–74, respectively. These two antigens were generated by the Proteomics Platform of the Centre de Recherche of the CHU de Québec. These peptides were injected at day 1, 43, 85, 127, 169, 211 in rabbits, at day 1, 29, 57, 92 in hamsters, and at day 1, 29, 57, 92, 146 in guinea pigs and rats. The amount of peptides injected was as follows: 400–650 μg for rabbits, 100–200 μg for rats and guinea pigs, and 100 μg for hamsters. The blood was collected prior to the first injection and 7 days post-injection. The peptides were diluted in phosphate-buffered solution (PBS) (in mM: 10.1 Na_2_HPO_4_, 1.8 KH_2_PO_4_, 136.9 NaCl, and 2.7 KCl, pH 7.4) and complete Freund’s adjuvant was mixed at a ratio 1:1 with peptides for the first injection. Two other peptides conjugated to keyhole limpet hemocyanin (KLH) that correspond to amino acid 87-101 (SYTSDPTQAGESLKS) and 390-404 (VEVSYPGQERWLRDY) were also injected six times (125–250 μg) in rabbit. The blood was collected 7 days post-injection. Recombinant purified protein produced in bacteria corresponding to amino acid 361–441 of mouse NTPDase8, named as peptide 76, was synthetized by the Molecular Biology and Production of Antibody Service of the Centre de Recherche du CHU de Québec. Rabbits were injected three times at day 1, 28, 56 and blood was collected 14 days after the second and the third injection.

### Polyclonal Antibodies by cDNA Immunization

Genetic immunization was carried out with plasmids encoding mouse NTPDase8 diluted in PBS 0.8×. Rabbits were injected with 1 mL of mouse NTPDase8 cDNA (0.65–0.8 mg/mL) consisting of 10 intradermic (ID) sites of 50 μL each and two intramuscular (IM) sites of 250 μL each. Hamsters were injected with 0.1 mL of mouse NTPDase8 cDNA (1 mg/mL) consisting of two ID sites of 25 μL each and one IM site of 50 μL. Guinea pigs and rats were injected with 0.2 mL of mouse NTPDase8 cDNA (1 mg/mL) consisting of two ID sites of 50 μL each and one IM site of 100 μL. Rabbits and hamsters were injected at day 1, 15, 29, 99, 183, 253, 286, 316, 384, and at day 1, 15, 29, 99, 170, respectively, rats and guinea pigs were injected at day 1, 15, 29, 99, 170, 283. The intradermal injections were done in the dorsal skin and the IM injections were done in the hind leg. The blood was collected prior to the first injection and between 12 and 14 days after the third and the subsequent injection.

### Monoclonal Antibody Production by cDNA Immunization

Hybridomas were generated in BALB/c mice by ID and IM injection with 100 μg of human NTPDase8 cDNA diluted in PBS 0.8× at day 1, 15, 29, 99, and 184. A final injection was made at day 297 using intact human embryonic kidney (HEK 293T) cells transfected with human NTPDase8 expression vector (see Cell Transfection and Western Blot). Spleen cells were collected 3 days after the prime boost cells injection and fusion with SP2/0 cells were done as previously described (Munkonda et al., 2009) with minor modifications. For this assay, the SP2/0 cells were combined with splenocytes at a ratio 1:5. The positive hybridomas were screened by enzyme-linked immunosorbent assay (ELISA). The hybridomas were cloned by limiting dilution and the produced immunoglobulins were purified on Protein A Sepharose CL-4B column as described (Munkonda et al., 2009).

### ELISA and Isotyping for Monoclonal Antibodies

ELISA plates (96 wells) were coated overnight (O/N) with 500 ng per well of lysates from African green monkey kidney (COS-7) cells transiently transfected with human NTPDase8 or untransfected diluted in PBS. After washing with PBS-Tween 0.05% (PBS-T), the wells were incubated for 1 h at 37°C in a blocking solution (0.5% bovine serum albumin diluted in PBS-T). After washing, the supernatant from each hybridoma was added to the well and incubated for 2 h at room temperature (RT), followed by four washing steps. Then a goat anti-mouse IgG (H + L)-HRP (1:2500) diluted in the blocking solution was incubated for 2 h at RT followed by four washing steps. The Enhanced K-Blue^®^ Substrate (Neogen Corporation, Lansing, MI, USA) was then added for 15 min and the reaction was stopped by the addition of an equal volume of 2 N sulphuric acid and absorbance at 450 nm was recorded. The isotype of the antibodies produced by each hybridoma was determined by a Mouse Immunoglobulin Isotyping ELISA Kit (BD Bioscience, Mississauga, ON, Canada) according to the manufacturer’s instruction. In brief, monoclonal rat anti-mouse IgG1, IgG2a, IgG2b, IgG3, IgA, and IgM were coated O/N in 96-well plates. After a washing step and a blocking treatment, each monoclonal anti-human NTPDase8 antibody was transferred to the wells. After washing steps, a rat anti-mouse Ig conjugated to HRP was added to each well, and revealed with a substrate provided in the kit. The plate was then read at 450 nm.

### Cell Transfection and Western Blot

COS-7 cells and HEK 293T cells were cultured and transiently transfected as indicated with mouse NTPDase8 or human NTPDase8 cDNA constructs as described previously (Kukulski et al., 2005). For Western blot assays, lysates from transfected or non-transfected COS-7 cells (6 μg, unless otherwise indicated) were resuspended in NuPAGE sample buffer, separated on NuPAGE 4–12% Bis-Tris gels under reduced or non-reduced conditions, as indicated, and transferred to an Immobilon-P membrane (Millipore, Bedford, MA, USA) by electroblotting according to the manufacturer’s recommendation. Membranes were then blocked with 2.5% non-fat milk in PBS containing 0.15% Tween20^®^(pH 7.4) O/N at 4°C and subsequently probed with the primary antibodies. Appropriate secondary HRP-conjugated antibodies were used, and the membranes developed with the Western Lightning^TM^ Plus-ECL system (PerkinElmer Life and Analytical Sciences, Waltham, MA, USA). In some experiments, a gel with a large sample well 6 cm long containing 120 μg of lysates, transferred and blocked as described above and then probed with antibodies using the Mini-Protean II multiscreen apparatus (Bio-Rad Laboratories Ltd., Mississauga, ON, Canada) in which 20 antibodies can be tested on one gel.

### Immunocytochemistry and Immunohistochemistry

Mouse liver was collected after animal perfusion with 4% PFA, tissues were then fixed in 4% PFA for 2 h and incubated O/N in 4% sucrose at 4°C and frozen in Tissue-Tek^®^O.C.T.^TM^ Compound (Sakura Finetek, Torrance, CA, USA). COS-7 cells or tissues sections (6 μm thick) of mouse liver were fixed in 10% phosphate-buffered formalin (Fisher Scientific, Ottawa, ON, Canada) mixed with cold acetone (Fisher Scientific, Ottawa, ON, Canada) and blocked in a PBS solution containing 7% normal goat serum for 30 min. COS-7 cells and tissue sections were incubated with the indicated primary antibody at 4°C. COS-7 cells and tissue sections were then treated with 0.15% H_2_O_2_ in PBS for 10 min to inactivate endogenous peroxidase, and with an avidin/biotin solution (Avidin/Biotin Blocking kit; Vector Laboratories, Burlington, ON, Canada) to prevent non-specific staining due to endogenous biotin. This step was followed by incubation with an appropriate biotin-conjugated secondary antibody at a dilution of 1:1000. The avidin–biotinylated HRP complex (VectaStain Elite ABC kit; Vector Laboratories) was added to optimize the reaction. Peroxidase activity was revealed with DAB as the substrate. Nuclei were counterstained with aqueous hematoxylin (Biomeda, Foster City, CA, USA) in accordance with the manufacturer’s instructions.

### Flow Cytometry (FACS)

HEK 293T cells transfected with mouse or human NTPDase8, were detached from the plates with a citric saline solution (135 mM potassium chloride, 15 mM sodium citrate). Samples of 2.5 × 10^5^ cells per tube were washed with an ice-cold PBS solution containing 1% FBS and 0.1% NaN_3_ [fluorescence-activated cell sorting (FACS) buffer] followed by incubation with the primary antibodies (serum from polyclonal anti-mouse NTPDase8 or purified monoclonal antibodies to human NTPDase8) or negative control (guinea pig preimmune sera or control mouse IgG2a (Sigma-Aldrich, Oakville, ON, Canada)) in FACS buffer for 1 h. After washes with FACS buffer solution, the cells were incubated with an appropriate Alexa-conjugated secondary antibody for 30 min on ice, washed with FACS buffer, and analyzed by flow cytometry (BD LSR II, BD Biosciences, San Jose, CA USA).

### Inhibition Assays

Inhibition assay were done as previously described (Munkonda et al., 2009) on cell lysates from human NTPDase8 transfected COS-7 cells at 37°C in two different buffers: modified Ringer buffer (120 mM NaCl, 5 mM KCl, 2.5 mM CaCl_2_, 1.2 mM MgSO_4_, 25 mM NaHCO_3_, 5 mM dextrose, 80 mM Tris–HCl, pH 7.4) and Tris/calcium buffer (5 mM CaCl_2_, 80 mM Tris–HCl, pH 7.4). After pre-incubation of the enzyme with the purified monoclonal antibodies to human NTPDase8 (5 μg/mL), substrate (ATP, 100 μM) was then added and incubated for 8–10 min, the reaction was then stopped with malachite green reagent. The inorganic phosphate released during the enzymatic reaction was measured as previously described with the malachite green assay (Baykov et al., 1988).

## Results

For many years we struggled to produce antibodies to mouse NTPDase8. We will present below our unsuccessful attempts that may help other groups to avoid these problems and improve their chances of success. We also present an efficient and convenient technique that we used to obtain excellent antibodies to several ectonucleotidases that are now available at ectonucleotidases-ab.com. In addition, we have tested the antibodies produced by different companies against mouse and human NTPDase8.

### Antibodies to Mouse NTPDase8

For the generation of mouse NTPDase8 antibodies, we first produced short peptides conjugated to KLH or in the form of MAPs. The number of animals tested for each antigen and conjugation type is indicated in **Table 1**. The MAPs was synthesized with peptide 829 and 830 corresponding to amino acid 1–14 and 60–74, respectively. These conjugated peptides and MAPs were injected in rabbits, hamsters, guinea pigs, and rats. The antiserum obtained for each animal was tested by Western blot under reduced condition. Note that all antisera generated from the immunization of peptides as an antigen were tested only by Western blot under denaturing conditions as this is the most likely technique that should work with such antigens. Generally, when no specific signal was obtained by Western blot with the sera from an animal immunized with peptides, no further experiments were done. The situation with intact and native proteins is different and will be described below. From all the sera with animals immunized with these MAPs, none of them gave a specific signal for mouse NTPDase8 (**Table 1** and data not shown).

**Table 1:** Antigen used and specificity of the antibodies to mouse and human NTPDase8.

Antigen	Conjugation	Host	Animals per group	Application				
				
				WB		IHC	ICC	FACS
							
				NR	Red.			
**Antibodies to mouse NTPDase8** (accession number: AY364442)					
Expression vector encoding mouse NTPDase8	None	Rabbit	3	–	–	–	NT	NT
		Rat	6	–	NT	–	+ (2/6)	NT
		Hamster	6	–	NT	–	–	NT
		Guinea pig	6	+ (5/6)	NT	–	+ (5/6)	NT
		Guinea pig	5	++	–	++	++	++
Peptide 829 (aa 1–14): MGLSWKERVFMALL	MAPs	Rabbit	2	–	–	–	NT	NT
		Rat	3	NT	–	NT	NT	NT
		Hamster	3	NT	–	NT	NT	NT
		Guinea pig	3	NT	–	NT	NT	NT
Peptide 830 (aa 60–74): QWPANKEKDTGVVSQ	MAPs	Rabbit	2	–	–	–	NT	NT
		Rat	3	NT	–	NT	NT	NT
		Hamster	3	NT	–	NT	NT	NT
		Guinea pig	3	NT	–	NT	NT	NT
Peptide 6768 (aa 87–101): SYTSDPTQAGESLKS	KLH	Rabbit	2	NT	+	NT	NT	NT
Peptide 6970 (aa 390–404): VEVSYPGQERWLRDY	KLH	Rabbit	2	NT	–	NT	NT	NT
Peptide 76 (aa 361–441)	None	Rabbit	3	NT	–	NT	NT	NT
Santa Cruz Biotechnologies (sc-160611)^1^	Unknown	Goat	N/A	NT	–	NT	NT	NT
**Antibodies to human NTPDase8** (accession number: AY430414)					
Expression vector encoding human NTPDase8	None	Mouse	9^2^ (4 mAbs)	++	++	++	NT	+
Aviva Systems Biology (ARP44815_P050) Peptide aa 110–159	Unknown	Rabbit	N/A	NT	–	NT	NT	NT


*Unless indicated otherwise with the number of reacting antisera over the total number of animals used for immunization, each animal in the same group reacted similarly in the experiments presented.*

*WB, Western blot; NR, non-reduced condition; Red., reduced condition; IHC, immunohistochemistry; ICC, immunocytochemistry; FACS, Fluorescence-activated cell sorting; NT, not tested; N/A, not applicable; aa, amino acid; mAbs, monoclonal antibodies. Results “-”, absence of reaction or same signal as in the negative control; “+”, weak positive reaction; “++”, strong and specific reaction and therefore antibody appropriate for research purposes. ^1^This antibody has been discontinued. ^2^For the production of monoclonal antibodies to human NTPDase8, nine BALB/c mice were immunized. Seven out of the nine mice gave a positive reaction on Western blot in non-reduced conditions. The mouse with the sera with the highest titer was used for the generation of monoclonal antibodies, as described in Section “Materials and Methods.” The four monoclonal antibodies obtained gave similar results for all experiments as indicated in the table.*

**FIGURE 1:**
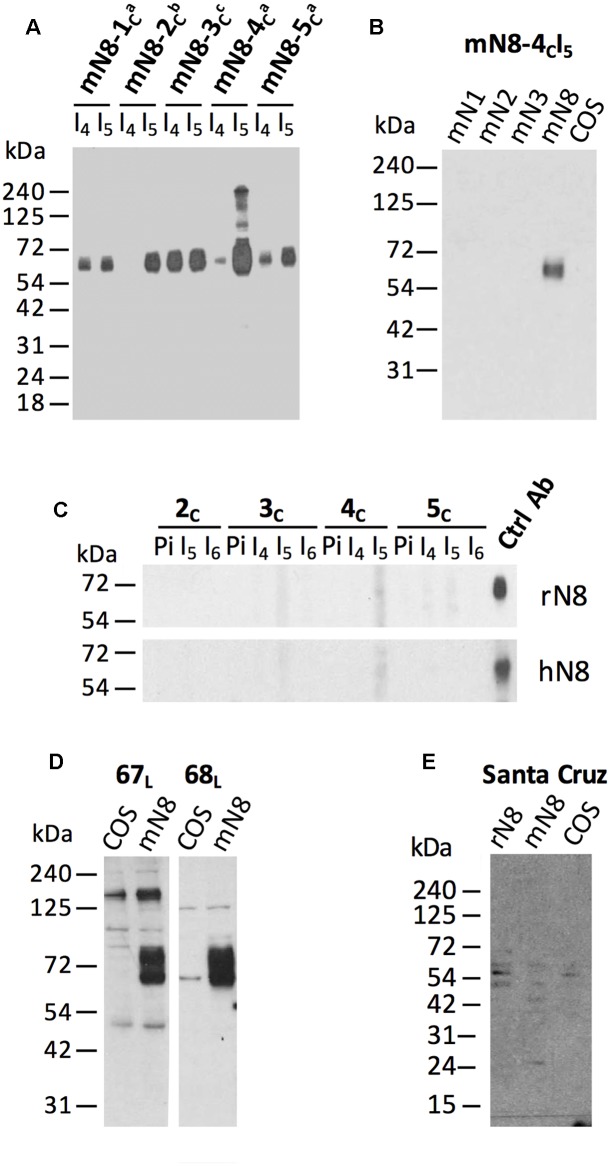
**Specificity of the polyclonal antibodies to mouse NTPDase8 by Western blot.** **(A)** Lysates (120 μg) for one large well from COS-7 cells transfected with mouse NTPDase8, were subjected to electrophoresis and probed with the five guinea pig (mN8-1*_c_*;-2*_c_*;-3*_c_*;-4*_c_*; -5*_c_*) antisera [bleeding after the fourth (I_4_) and fifth (I_5_) injection] against mouse NTPDase8 at different dilution (a: 1:1000; b: 1:500; c: 1:2000). **(B)** NTPDases specificity tested on lysates from COS-7 cells transfected with mouse NTPDase1 (mN1), -2 (mN2), -3 (mN3), -8 (mN8) or from non-transfected COS-7 cells (COS) and probed with the guinea pig anti-mouse NTPDase8 mN8-4*_c_*I_5_ at a dilution of 1:1000. **(C)** Species specificity tested on lysates (120 μg; large well) of COS-7 cells transfected with either rat (rN8) or human (hN8) NTPDase8 and incubated with four guinea pig antisera corresponding to the bleedings after the fourth (I_4_), the fifth (I_5_) and the sixth (I_6_) injection or with the preimmune serum at a dilution of 1:500. Control antibodies to rat NTPDase8, rN8-8*_c_*I_5_ (1:1000) (previously described by Fausther et al., 2007) and to human NTPDase8, hN8-C5_s_ (0.2 μg/mL) were used to confirm the presence of rat or human NTPDase8 protein in COS-7 lysates. **(D)** Lysates from COS-7 cells transfected with mouse NTPDase8 expression vector (mN8) or non-transfected (COS) and incubated with either mN8-67_L_I_5_ or mN8-68_L_I_6_ antiserum diluted 1:500 from rabbits immunized with peptide 6768 conjugated to KLH. **(E)** Lysate from COS-7 cells transfected with mouse (mN8) or rat (rN8) NTPDase8 or non-transfected (COS) and incubated with a commercial anti-mouse NTPDase8 (1:200) from Santa Cruz Biotechnology Inc. (Dallas, TX, USA). All gels were run under non-reduced conditions except in panel **(D)** and **(E)** where the protein samples were treated with β-mercaptoethanol.

Two other peptides conjugated to KLH, were injected in rabbits. The antisera from the rabbits injected with the peptide corresponding to amino acid 87–101 of mouse NTPDase8 showed a signal on lysate of mouse NTPDase8 transfected cells in Western blot (**Figure 1D**). Due to high level background, these antibodies were not further used in other techniques. No specific signal was obtained with the sera of rabbits injected with the KLH conjugated peptide corresponding to amino acid 390–404 (data not shown).

The service of Molecular Biology and Production of Antibody Service of the Centre de Recherche du CHU de Québec produced in bacteria a mouse NTPDase8 recombinant section from amino acid 361 to 441 (peptide 76). The peptide was purified by affinity chromatography on nickel-nitrilotriacetic acid resin, dialyzed and injected in rabbits. Again, none of the antisera obtained detected mouse NTPDase8 by Western blot in reduced condition (data not shown). Note that the peptides tested above were selected following *in silico* analysis using different programs to determine the hydrophobicity, antigenicity and sequence homology such as Peptool (BioTools Incorporated, Edmonton, AB, Canada) and Kyte and Doolittle hydrophobicity scale. The N-terminal region generally known to represent a good choice for immunization was also synthetized. Unfortunately, little success was obtained with these peptides as detailed above.

Injections of mouse NTPDase8 cDNA were then used in rabbits, hamsters, rats, and guinea pigs. The serum obtained for each animal was tested by Western blot. As the antigen here is the complete native form of mouse NTPDase8 produced by the host cells, the Western blots were first carried out in non-reducing conditions. No specific staining was obtained either with the rabbits, the hamsters or the rats (data not shown). On the other hand, the antisera developed by guinea pigs gave a weak signal on lysate from mouse NTPDase8 transfected COS-7 cells (data not shown). Of all the techniques used and from all species tested, the guinea pigs injected with cDNA was the technique and the species from which the best results were obtained. As the antisera of the first group of guinea pigs were positive but not sufficiently strong to work with, we repeated with a second series of five guinea pigs with the cDNA immunization technique. The sera of those last 5 guinea pigs gave a strong positive signal in Western blot with different intensity levels at the right molecular weight (**Figure 1A**). The detected band in Western blot for mouse NTPDase8 appears higher than the calculated molecular weight (54,650 Da) due to eight potential N-linked glycosylation sites. We do not have an explanation for the difference in immunoreactivity between the first groups of guinea pigs and the second group.

The cross-reactivity of the two best antibodies (mN8-3*_c_*I_5_ and mN8-4*_c_*I_5_) was tested on lysates of COS-7 cells transfected with other closely related members of the mouse E-NTPDase family as well as with non-transfected cells. **Figure 1B** shows that the antiserum mN8-4*_c_*I_5_ does not recognize mouse NTPDase1, -2, -3 or any other proteins from non-transfected COS-7 cells. Similar results were obtained for the other antibody tested: mN8-3*_c_*I_5_ (data not shown). The specificity was also confirmed on recombinant NTPDase8 from rat and human species, as shown on **Figure 1C**, no antiserum after the fourth (I_4_), the fifth (I_5_) and the sixth injection (I_6_) of those guinea pigs (mN8-2*_c_*; -3*_c_*; -4*_c_*; -5*_c_*) cross-reacted with these two species. Similar results were obtained with mN8-1*_c_* on rat NTPDase8 (data not shown). These four guinea pig antisera (mN8-2*_c_*; -3*_c_*; -4*_c_*; -5*_c_*) were also tested in reduced conditions against mouse NTPDase8 but none of them recognized the protein reduced with β-mercaptoethanol (data not shown).

**FIGURE 2:**
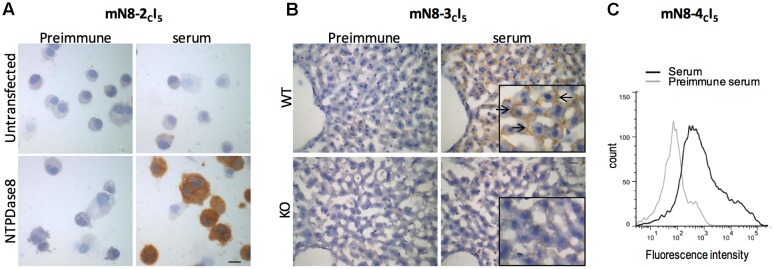
**Specificity of the best antisera for immunocytochemistry, immunohistochemistry, and flow cytometry.** **(A)** Immunocytochemistry of COS-7 cells transfected with mouse NTPDase8 (lower panels) or non-transfected COS-7 cells (upper panels) were probed with the guinea pig anti-mouse NTPDase8 mN8-2*_c_*I_5_ (right panels), or with its preimmune serum (left panels), both at a 1:250 dilution. **(B)** Immunohistochemistry on a liver deficient in NTPDase8 expression (lower panels) or on a wild type (WT) mouse liver (upper panels) incubated with the preimmune serum (left panels) or the antiserum mN8-3*_c_*I_5_ (right panels) at a dilution of 1:500. Insets are 2× magnifications. Arrows show stained canaliculi. **(C)** Flow cytometry on mouse NTPDase8 transfected HEK 293T cells incubated with the mN8-4*_c_*I_5_ antiserum or its preimmune serum both at 1:80 dilution. In panels **(A)** and **(B)**, counterstaining of nuclei (blue) was performed with aqueous hematoxylin. Scale bar 25 μm.

We next tested whether the best antisera on Western blot could immunolocalize NTPDase8 by immunocytochemistry and immunohistochemistry. The antisera mN8-2*_c_*I_5_ gave a strong positive signal on cells transfected with an expression vector encoding mouse NTPDase8 (**Figure 2A**, lower right panel). The absence of staining in non-transfected COS-7 cells confirmed the specificity of the reaction (**Figure 2A**, upper right panel). In addition, no signal could be detected with the preimmune serum either on non-transfected or NTPDase8 transfected COS-7 cells (**Figure 2A**, left panels). Similar results were obtained with the mN8-3*_c_*I_6_ and mN8-4*_c_*I_5_ antiserum (data not shown).

These antisera were also efficient and specific to detect the native NTPDase8 by immunohistochemistry, as evaluated by use of wild type and knockout mouse for *Entpd8* gene. We previously demonstrated the presence of NTPDase8 in rat (Fausther et al., 2007) and porcine liver canaliculi (Sévigny et al., 2000). We have also detected the presence of mRNA in mouse liver (Bigonnesse et al., 2004) suggesting that this protein may also be found in the same structure in mouse. As expected, mN8-3*_c_*I_5_ antiserum stained the canaliculi in the wild type mouse liver but not in the NTPDase8 knockout mouse (**Figure 2B**). Similar results were obtained with mN8-1*_c_*I_5_; -2*_c_*I_5_; -4*_c_*I_5_; -5*_c_*I_5_ antiserum (data not shown).

Each of the last five guinea pig anti-mouse NTPDase8 antisera was also efficient in flow cytometry. Indeed, they showed a shift in fluorescence intensity on cells transfected with a mouse NTPDase8 expression vector when compared with the preimmune serum (**Figure 2C** for mN8-4*_c_*I_5_ and data not shown for the other guinea pig antisera). Overall, these data indicate that the guinea pig antibodies detect the native mouse NTPDase8 protein.

In contrast to the immunization techniques that use native proteins as a source of antigen, as in the cDNA immunization technique with the full gene encoded as we did here, the commercial anti-mouse NTPDase8 from Santa Cruz Biotechnology Inc. was generated against a synthetic polypeptide. Therefore, if it can detect mouse NTPDase8 it must do in Western blot in denaturing and reduced conditions. In these conditions, the same non-specific bands were detected in both, lysates from transfected cells with mouse NTPDase8 and from non-transfected cells (**Figure 1E**).

### Antibodies to Human NTPDase8

Hybridomas were generated from B-cells of BALB/c mice injected with human NTPDase8 cDNA. As our previous assays using only cDNA immunization were ineffective for some proteins (data not shown), we have made the final boost with intact HEK 293T cells transfected with the same human NTPDase8 expression vector to increase the titer and our chances of success in obtaining hybridomas. Even if the background would be expected to increase in the sera, this is not an issue with monoclonal antibodies. With this technique, we obtained four hybridomas by limiting dilution named hN8-B3_s_, hN8-C5_s_, hN8-D7_s_, and hN8-D7A_s_, which produced a positive response in ELISA on lysates of human NTPDase8 transfected cells, and a negative signal with lysates from non-transfected cells. The specificity of these monoclonal antibodies was tested by Western blot, ELISA, and flow cytometry.

**FIGURE 3:**
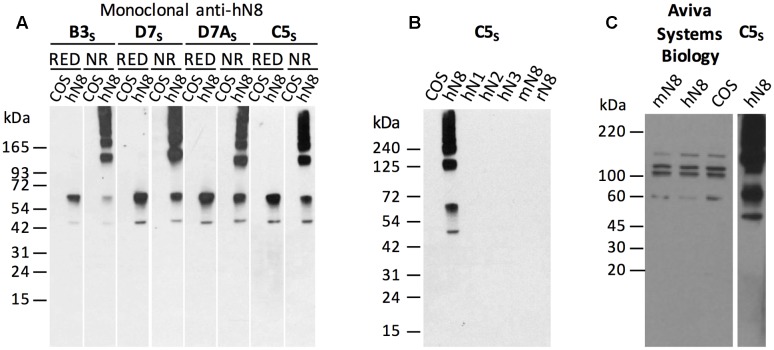
**Specificity of the mouse monoclonal antibodies to human NTPDase8 by Western blot.** **(A)** Western blotting using lysates from COS-7 cells transfected with human NTPDase8 (hN8) or non-transfected cells (COS) in reduced (RED) or in non-reducing conditions (NR) were incubated separately with each anti-human NTPDase8 hybridoma, hN8-B3_s_, -D7_s_, -D7A_s_, and -C5_s_, as indicated. **(B)** Specificity of hN8-C5_s_ (0.5 μg/mL) antibody on lysates from COS-7 transfected with human NTPDase1 (hN1), -2 (hN2), -3 (hN3), -8 (hN8), mouse NTPDase8 (mN8), rat NTPDase8 (rN8) or non-transfected cells (COS) in non-reduced condition. **(C)** A commercial anti-human NTPDase8 antibody from Aviva Systems Biology tested on lysates from COS-7 transfected with mouse (mN8) or human (hN8) NTPDase8 as well as non-transfected cells (COS) in reduced condition (left panel). A positive control in non-reduced conditions on a human NTPDase8 COS-7 cell lysate probed with hN8-C5_s_ antibody was performed on the same gel. Additional note. Panels **(A)** and **(B)** represent two gels performed in the same time. The first 12 lanes of panel **(A)** constitute the first gel and the last four lanes were part of the gel presented in panel **(B)**. The last two lanes of panel **(A)** (COS and hN8) are reproduced in the first two lanes of panel **(B)**. Each demarcation shown in panels **(A)** and **(C)** represents a lane with non-reduced COS lysates used to stop diffusion of β-mercaptoethanol. These lanes were cut out of the data presented.

The four hybridomas were tested in Western blot on lysates from COS-7 cells transfected with human NTPDase8 or non-transfected cells in reduced and non-reduced condition. As demonstrated in **Figure 3A**, human NTPDase8 antibodies recognize human NTPDase8 proteins in both reduced and non-reduced forms, suggesting that the higher bands observed over 65 kDa in the non-reduced samples are due to multimer formation. Indeed, none of these higher molecular weight bands are detected in the control sample proteins. Human NTPDase8 have a calculated molecular weight of 53,773 Da plus seven potential glycosylation sites for about 2–4 kDa each. In addition, the specificity of these four hybridomas was also tested in Western blot on other human NTPDases (NTPDase1, -2, -3) and on other NTPDase8 species (mouse and rat) using lysates from transfected COS-7 transfected as well as from non-transfected cells. As illustrated in **Figure 3B**, the antibody named hN8-C5_s_ detected only human NTPDase8. No cross-reaction was observed with human NTPDase1, -2, -3 nor with the mouse or rat NTPDase8 showing that these antibodies are highly specific. Similar results were obtained for the three other antibodies.

The isotype of the antibodies produced by these hybridomas were determined by ELISA using rat monoclonal antibodies specific for different mouse immunoglobulin which revealed that all four hybridomas are IgG2a (data not shown).

**FIGURE 4:**
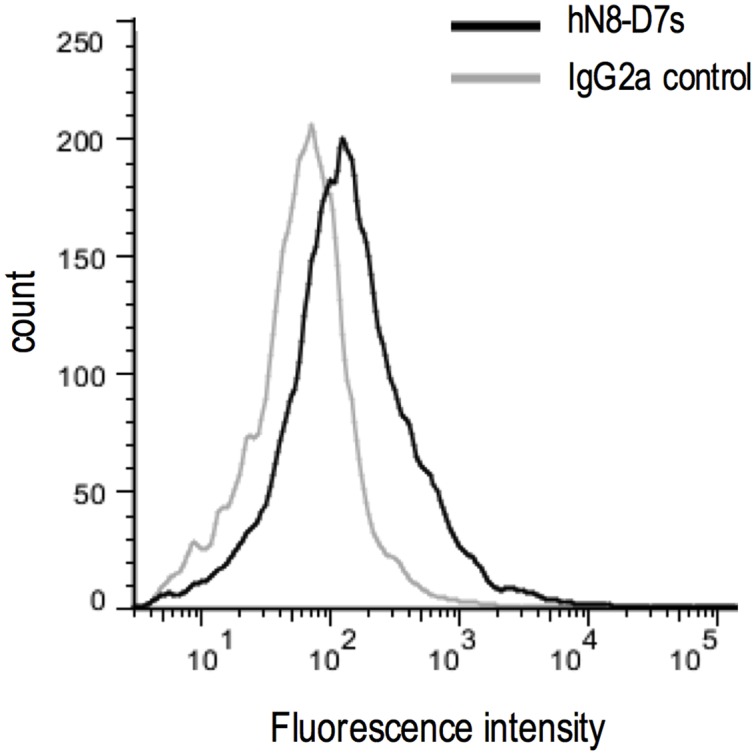
**Specificity of hN8-D7_S_ by flow cytometry.** Flow cytometry on human NTPDase8 transfected HEK 293T cells and incubated with an anti-human NTPDase8 (hN8-D7_s_) or its isotype control both used at 10 μg/mL.

The four purified hybridomas anti-human NTPDase8 were tested by flow cytometry on human NTPDase8 transfected HEK 293T cells. All hybridomas produced a shift in the fluorescence at different intensity; the best antibody for this technique was hN8-D7s (diluted at 10 μg/mL; **Figure 4**), data not shown for the three other monoclonal antibodies.

These four monoclonal antibodies were also tested in enzyme activity inhibition assays but none of them were able to inhibit human NTPDase8 (data not shown).

The commercial anti-human NTPDase8 (Aviva Systems Biology, San Diego, CA, USA) was tested by Western blot. No specific staining at the expected molecular weight could be detected in reduced conditions. As seen in **Figure 3C**, the bands obtained were the same in the positive samples [COS-7 cells transfected with human NTPDase8 (hN8)] and in the negative control [non-transfected COS-7 cells (COS)].

## Discussion

In the present work, we have generated polyclonal antibodies against mouse NTPDase8 and four hybridomas that produce IgG2a against human NTPDase8. Many experimental approaches were necessary to generate antibodies against mouse NTPDase8, indeed, even if five different peptides were used to produce antibodies against mouse NTPDase8 injected in different hosts, and conjugated differently, only the peptides corresponding to amino acid 87–101 conjugated to KLH injected in rabbits gave a positive band on Western blot. The immunization with an expression vector encoding for the full coding sequence of mouse NTPDase8 in rabbit, guinea pig, rat, and hamster gave successful result only in guinea pigs (**Table 1**). This technique was also useful for the production of antibodies against several other ectonucleotidases (Sévigny et al., 2002; Bartel et al., 2006; Vekaria et al., 2006; Fausther et al., 2007, 2012; Martín-Satué et al., 2009). The guinea pig anti-mouse NTPDase8 antibodies were validated in different techniques. They are specific and efficient for immunohistochemistry, for immunocytochemistry, for flow cytometry as well as for Western blot but only in non-reduced conditions, suggesting that the epitope is discontinuous and part of a tertiary structure of the protein involving disulphide bridges. These antibodies were very specific to mouse NTPDase8 and they did not cross-react with cells transfected with the other NTPDases that show the highest homology to NTPDase8, between 39 and 44% identity, namely NTPDase1, -2, and -3 (Bigonnesse et al., 2004; Lavoie et al., 2004), or with NTPDase8 from rat and human species. The other NTPDases which are more different in structures and in amino acid sequence identity (lower than 25% identity to mouse NTPDase8) were not tested. All the difficulties that we encountered to produce antibodies to mouse NTPDase8, especially those using synthetized peptides, suggests that this protein is poorly immunogenic.

In addition, the antibody against mouse NTPDase8 allowed the localization of NTPDase8 in mouse liver canaliculi as we previously observed for rat NTPDase8 (Fausther et al., 2007).

To generate monoclonal antibodies against human NTPDase8, an injection of human NTPDase8 transfected HEK 293T cells at the last boost after several cDNA immunizations was determined to be an efficient technique. The monoclonal antibodies against human NTPDase8 were also validated with different techniques. Our results show that these monoclonal antibodies are efficient in all techniques tested: Western blot (reduced and non-reduced conditions), indirect ELISA, immunohistochemistry (data not shown) and flow cytometry (**Table 1**). The four hybridomas may originate from the same original B cell clone since all experiments performed yielded similar results with each of the four monoclonal antibodies and that all are IgG2a.

In addition, we also tested commercial antibodies to mouse and human NTPDase8. Neither of them detected a specific band on Western blot under the conditions used in our experiments. Indeed, the same background bands could be seen in both COS-7 extracts from cells overexpressing NTPDase8 and in COS-7 extract controls (**Figures 1E**, **3C**). The commercial antibodies were used at the concentration recommended by the companies. It is noteworthy that we allowed an overreaction to detect any minor bands. As a comparative control which is illustrated in **Figure 3C**, the amount of cell lysates loaded, containing human NTPDase8, and time of reaction resulted in an intense band with the hN8-C5_s_ antibody. Similar results were obtained when comparing the reaction obtained with mN8-4*_c_*I_5_ antisera with the antibody from Santa Cruz (**Figure 1E** and data not shown).

## Conclusion

In this work, we generated specific antibodies to mouse and human NTPDase8 with very convenient cDNA immunization techniques. These antibodies are useful for Western blot, ELISA, immunohistochemistry, immunocytochemistry, and flow cytometry. These antibodies allowed the immunolocalization of NTPDase8 in mouse liver canaliculi. It is noteworthy that the commercial antibodies tested here against mouse or human NTPDase8 failed to give a specific reaction. This reiterates previously published results evaluating other commercially available antibodies, which indicate that commercial antibodies need to be carefully validated before they can be correctly and effectively used, e.g., for antibodies against angiotensin II AT_2_ (Hafko et al., 2013), P2Y_6_ (Yu and Hill, 2013), and alpha1-adrenergic receptor subtypes (Jensen et al., 2009).

## Author Contributions

JP performed Western blot, immunocytochemistry, activity tests, flow cytometry, and wrote the first draft of the manuscript; MS did immunohistochemistry assays; JL prepared the protein extracts; MF performed immunohistochemistry, immunocytochemistry, and Western blot; FB performed immunohistochemistry and immunocytochemistry; JS supervised the study.

## Disclaimer

The monies received to obtain the antibodies were reinvested into antibody generation and research.

## Conflict of Interest Statement

The authors declare that the research was conducted in the absence of any commercial or financial relationships that could be construed as a potential conflict of interest.
